# The differential expression of MC1R regulators in dorsal and ventral quail plumages during embryogenesis: Implications for plumage pattern formation

**DOI:** 10.1371/journal.pone.0174714

**Published:** 2017-03-29

**Authors:** Thanh-Lan Gluckman, Nicholas I. Mundy

**Affiliations:** 1 Department of Zoology, University of Cambridge, Downing Street, Cambridge, United Kingdom; 2 Center for Interdisciplinary Research in Biology, Collège de France, Paris, France; Northwestern University, UNITED STATES

## Abstract

Melanin pigmentation patterns are ubiquitous in animals and function in crypsis, physical protection, thermoregulation and signalling. In vertebrates, pigmentation patterns formed over large body regions as well as within appendages (hair/feathers) may be due to the differential distribution of pigment producing cells (melanocytes) and/or regulation of the melanin synthesis pathway. We took advantage of the pigmentation patterns of Japanese quail embryos (pale ventrum and patterned feathers dorsally) to explore the role of genes and their transcripts in regulating the function of the melanocortin-1-receptor (MC1R) via 1. activation: pro-opiomelanocortin (POMC), endoproteases prohormone convertase 1 (PC1) and 2 (PC2), and 2. inhibition—agouti signaling and agouti-related protein (*ASIP* and *AGRP*, respectively). Melanocytes are present in all feather follicles at both 8 and 12 days post-fertilisation (E8/E12), so differential deposition of melanocytes is not responsible for pigmentation patterns in embryonic quail. *POMC* transcripts expressed were a subset of those found in chicken and *POMC* expression within feather follicles was strong. *PC1* was not expressed in feather follicles. *PC2* was strongly expressed in all feather follicles at E12. *ASIP* transcript expression was variable and we report four novel *ASIP* transcripts. *ASIP* is strongly expressed in ventral feather follicles, but not dorsally. *AGRP* expression within feather follicles was weak. These results demonstrate that the pale-bellied quail phenotype probably involves inhibition of MC1R, as found previously. However, quail may require *MC1R* activation for eumelanogenesis in dorsal feathers which may have important implications for an understanding of colour pattern formation in vertebrates.

## Introduction

Pigmentation patterns are common in mammals and birds and require precise spatiotemporal control during development. Patterning can involve regional variation in hair/feather pigmentation over the body and/or patterning within individual hairs or feathers. In birds, the only known type of pigmentation that can be precisely controlled to produce differential pigmentation within-feathers is melanin, which is produced by melanocytes [1]. Two mechanisms of pigment pattern formation in feathers have been proposed: melanocyte distribution/differentiation and pigment-type switching via the melanocortin-1 receptor (MC1R). An absence of mature melanocytes underlies the unpigmented white bars in the feathers of some chicken breeds [2] and it may be that the black at hatch locus (*Bh*) is involved in longitudinal stripe formation on the dorsum of quail [3]. However, these mechanisms cannot explain patterning involving phaeomelanin-eumelanin differences which are thought to rely on differential regulation of MC1R activity [2,4–6].

The MC1R is a G-protein coupled receptor expressed in the plasma membrane of melanocytes [7]. Activation of MC1R leads to eumelanin synthesis whereas inhibition of MC1R leads to phaeomelanin synthesis, or an absence of melanin synthesis. Three factors affect the level of MC1R activation: the concentration of extracellular MC1R agonists such as alpha melanocyte stimulating hormone (α-MSH), the concentration of extracellular MC1R antagonists/inverse agonists such as agouti signaling protein (ASIP), and the intrinsic basal level of activity of the receptor itself. The importance of the latter is seen in the large number of mutations in *MC1R* in birds, mammals and other vertebrates that can lead to gross changes in coloration over the body (e.g. [8–10]). In addition, the basal level of activity in wildtype MC1R varies among vertebrate species, e.g. mice have much higher basal MC1R activity than humans [11]. This is reflected in the phenotypes of *POMC* null mutants, in which MC1R stimulation is absent: such mutants have little effect on eumelanin synthesis in mice whereas *POMC* mutations can sometimes lead to red hair in humans, although this is variable [12–15].

For pigment patterning, antagonism of MC1R by ASIP has received the most attention. In mammals, *ASIP* has been implicated in dorso-ventral patterning, temporal-specific regulation of pigmentation during hair growth leading to banding patterns and inhibition of melanocyte differentiation [16–19]. The dorso-ventral and temporal-specific regulation of *ASIP* expression occurs via different promoters in mice [16,17,20]. In adult chicken and quail, *ASIP* may function in dorso-ventral patterning, within-feather patterning and sexual dichromatism [4,6,21]. Birds also have multiple alternatively spliced transcripts of *ASIP*, with some evidence to suggest that the distal promoter site is also ventral specific [4,21] but overall their relationship to pattern function in birds is poorly understood compared to mammals. Given the function of agouti in mouse coat colour, it was postulated that the *ASIP* paralogue agouti-related protein (*AGRP*) may have a role in avian plumage patterning [22,23]. *AGRP* encodes a melanocortin receptor antagonist and its gene structure is conserved between mammals and birds [24,25]. Unlike in mammals, *AGRP* is expressed in the skin in birds [26] but its potential role in pigmentation patterning has yet to be investigated.

The most important class of MC1R agonists are melanocortin peptides derived from the precursor pro-opiomelanocortin (POMC). The main melanocortins implicated in pigmentation are α-MSH and adrenocorticotropic hormone (ACTH) [5,27,28]. In the chicken, ACTH binds with a higher affinity to MC1R than MSH and it has been proposed that ACTH may have a function in pigmentation patterns [5,11]. The endoproteases prohormone convertase 1 (PC1), encoded by *PCSK1*, and prohormone convertase 2 (PC2), encoded by *PCSK2*, are responsible for cleavage of POMC and its products. PC1 cleaves the POMC peptide to make ACTH as well as β-lipotropin whereas PC2 cleaves POMC or ACTH to make α-MSH or desacetyl-α-MSH [11,29,30]. In tawny owls (*Strix aluco*), it was suggested that POMC/PC1/PC2 may mediate a correlation between melanin-based coloration and fitness [31]. Therefore, the loci involved in activation of MC1R may have an important role in feather patterning.

In a study of extracellular MC1R ligands in the silky and Okayama-Jidori breeds of chicken, it was shown that POMC, PC1 and PC2 are expressed within adult feather follicles [5]. Clear positive signals were found for ACTH using dot-blotting, but MSH was only expressed at trace levels. Investigating patterns of expression in adult feather follicles, four alternatively spliced *POMC* transcripts were detected that are transcribed from two promoter sites (Class a and b), either with or without a non-coding exon [5].

**Fig 1:**
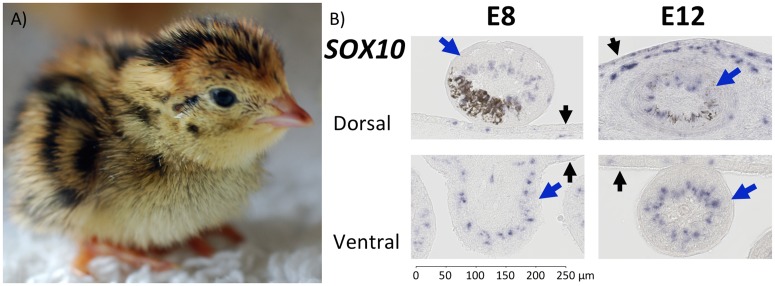
Japanese quail (*Coturnix japonica*) chick and melanocyte distribution at embryonic stages E8 and E12. A) The dorsal pigmentation of *C*. *japonica* comprises alternating stripes of dark and light feathers that are uniformly pigmented with one type of melanin, as well as feathers that are pigmented with both eumelanin and pheomelanin. Photo by Cowgirl Jules / CC BY 2.0. B) *In situ* hybridization for *SOX10* to determine melanocyte distribution in Japanese quail feather follicles during embryogenesis at E8 and E12. Feather follicles are labelled with a large blue arrow and the epidermis with a small black arrow. At E8 and E12 eumelanin is rarely present in ventral feather follicles but is frequently observed in feather follicles on the dorsal surface. Melanocytes are present within the epidermis and feather follicles. Staining of *SOX10* occurs in areas where individual barbs are developing in feather follicles on both the ventral and the dorsal surface of E8 and E12 quail embryos. The distribution of melanocytes is not restricted to areas pigmented with melanin but also occurs where melanin is absent.

The development of plumage pigmentation in Japanese quail (*Coturnix japonica*) is a classic model system [e.g. 32–34]. Quail embryos develop a pale (phaeomelanin) belly, and dorsal stripes of dark (eumelanin) and pale feathers in addition to feathers patterned with both eumelanin and phaeomelanin (Fig 1a). In adulthood, Japanese quail have pronounced patterns on the dorsal surface and retain the pale-bellied phenotype.

Previous studies on the function of *ASIP* and *POMC* in bird plumage patterns were conducted in adults and did not control well for developmental stage, potentially resulting in an under-estimation of the contribution of these ligands [4–6,21]. In this study, we examined the relative contribution of melanocyte differentiation, MC1R inhibition and MC1R stimulation to the species-typical plumage. We found that melanocytes are distributed throughout feather follicles on the dorsal and ventral surface at E8 and E12. A gene involved in down regulating the *MC1R* pathway (*ASIP*) was restricted to the ventral surface and genes involved in upregulating the *MC1R* pathway (*POMC*, *PCSK2*) were expressed in all feather follicles on the ventrum and dorsum at E8 and E12 stages of development.

## Materials and methods

Fertilized wild-type Japanese quail (*Coturnix japonica*) eggs were obtained from commercial sources. Feather pigmentation is first visible between 8–9 days, the equivalent of chicken stages 35–36, and is fully developed by 11–12 days [35]. Therefore, we harvested embryos at E8 and E12. As there can be variation in developmental stage between individuals, each embryo was checked to ensure that it was at the required stage of development. Embryos where the eyelid had begun to overgrow the surface of the eyeball and had some feather pigmentation on the dorsal surface, but not on the forehead and crown, were considered to be representative of E8. Embryos that had prominent pigmentation and white feather germs around the eye were considered representative of E12. Some of the E12 embryos did not have pigmentation on the feet, a key spotting character. However, all specimens had fully developed plumage and were considered representative of well-developed embryonic plumage appropriate for E12. Tissue and embryos were harvested in phosphate buffered saline (PBS) containing diethylpyrocarbonate (DEPC) treated- distilled water. Samples of ventral and dorsal epidermal tissue containing skin and feather follicles were dissected and stored separately in RNAlater at 4 degrees overnight for increased tissue penetration, and then at -20 until RNA extraction. For RT-PCR, we sampled three embryos of developmental stage E8 and three embryos of developmental stage E12, and for *in situ* hybridization we sampled 2–3 individuals of each of these developmental stages.

### RT-PCR analysis

Total RNA was extracted from each sample with an RNeasy mini-kit (Qiagen), with a final elution in 30μl of RNase free distilled water. RNA integrity, purity and concentration (RIN values) were quantified using a BioAnalyser (Agilent) and only extractions with a RIN value of > = 8 were retained for further analysis. First strand cDNA syntheses were conducted with 1–3μg RNA and 1μl of 150ng/μl N6 primer in a total volume of 20μl using Superscript RT II (Invitrogen) following the manufacturer’s instructions.

We searched for transcripts of our genes of interest in quail and chicken on Genbank. Where possible, we used existing primers documented in other studies. Alternatively spliced transcripts and/or coding regions were amplified using published primers and primers designed from available quail and chicken gene sequences from Genbank for *ASIP*, *AGRP*, *POMC*, *PCSK1* and *PCSK2* (Table A in S1 File). Several novel *ASIP* transcripts were found, and these were fully sequenced using new internal primers (Table A in S1 File). For *POMC*, we used published chicken alternatively spliced transcripts [5] and unpublished quail transcripts from Genbank (accession number AB620012.1, AB620012.1, AB620013.1). All of the isoforms expressed in this study contained a unique non-coding exon and primers were designed to be isoform specific. Where available quail coding sequences were relatively short in comparison to the chicken, we designed primers using chicken transcripts to ensure increased sequence coverage.

Reverse transcription polymerase chain reaction (RT-PCR) was carried out on cDNA derived from the ventral and dorsal surface of each embryo (as described above) to examine which transcripts of our genes of interest were expressed in each stage of development. RT-PCR was performed in 25μl total reactions containing 1.0 unit BIOTAQ polymerase (Bioline) 1.5mM MgCl_2_, 0.2μl 50mM dNTP, 1μl 10μM each primer and 1μl of first strand synthesis product. PCR reactions were performed in a DNA Engine (MJ Research, Watertown, MA), with the following cycling parameters: Heated lid 110°C, 94°C for 2 mins.; 40 x 94°C for 30 secs., 55–64°C for 30–60 secs., 72°C for 1 min; 72°C for 5 mins. As described previously, primers were designed to be isoform specific resulting in a single band in our RT-PCR experiments. Sequencing was performed with Sanger sequencing on both strands using the PCR primers.

New sequences were assembled and edited using the SeqMan software (DNASTAR Inc., Madison WI, USA). Alignments were performed in Mega 6.06 [36]. We used the housekeeping gene *β-actin* as a positive control for all RT-PCR reactions. Accession numbers of sequences from which the primers were designed, and amplicon size are listed in Table A in S1 File. All new sequence data have been deposited in Genbank with the following accession numbers: KR873138- KR873141.

### *In situ* hybridization

Tissue preparation and procedures used DEPC-H_2_0 and DEPC-PBS as necessary. For *in situ* hybridization, whole embryos were fixed in modified Carnoy’s (60% ethanol, 11.1% formaldehyde, 10% glacial acetic acid) for four hours, dehydrated in ethanol and cleared in Histosol (National Diagnostics). E12 embryos were cut along the sagittal plane to improve Histosol tissue penetration. Embryos were embedded in paraffin wax (Raymond Lamb) and transversely sectioned with a rotary microtome (Micron) between the forelimbs and hindlimbs where the dorsal stripes are strongly expressed—herein referred to as dorsal for brevity. E8 embryos were sectioned at 10μm. Because of the size difference and the extended time required to clear the E12 embryos in Histosol (3 weeks), the E12 embryos were more prone to crumbling and were sectioned at 14μm, which is still adequate for probe tissue penetration as shown in the positive controls. Eight serial sections per embryo per slide were mounted in DEPC-H_2_0 on Superfrost^®^ Plus slides (VWR), and dried overnight at 37°C.

All experiments represent a minimum of two replicates of E8 and E12 for each probe and the experiments were considered representative of development where most sections in each slide showed the same result—sections with background or abnormal staining that covered most of a section were excluded from the final interpretation or repeated if the probe required optimisation. For each round of *in situ* hybridization experiments, at least one embryo was represented twice, one had the sense probe, and other replicate had the anti-sense probe. Other than the probe applied, the slide with the sense probe was experimentally tested in the same way as the corresponding slide with the anti-sense probe, e.g. the colour reaction was applied for the same amount of time at the same temperature.

We used *SOX10* as a marker to examine melanocyte distribution. *SOX10* plasmids, containing the full-length chicken 2.2kb *SOX10* sequence, were gifts of M. Bronner (Caltech, Pasadena, CA) to C.V.J Baker. For all other probes, primers for the coding sequences used to generate each probe are listed in Table A in S1 File. PCR product was ligated into One Shot Top10 chemically competent cells (Invitrogen) using a PCR cloning kit (Qiagen). PCR products were incubated with cloning reagents and the vector overnight at 4°C in a total volume of 5 μl comprising 2.5μl 2x Ligation Master mix =, 2μl PCR product =, and 0.5μl pDrive cloning vector. =. Cells were transformed and grown according to the manufacturer’s instructions (Invitrogen). Plasmids were extracted using alkaline denaturation and stored at -20°C until probe synthesis [37]. To examine the direction of the insert relative to the promoter sites, plasmids were sequenced with Sanger sequencing. Probes were linearised using standard restriction enzymes and transcribed with DIG RNA labeling mix (Roche). The *SOX10* anti-sense probe was linearized with HindIII and transcribed from T3. The *ASIP*, *PCSK1*, and *PCSK2* anti-sense probes were linearized with NotI and transcribed from SP6, and the sense (negative control) probes were linearized with PstI and transcribed from T7. The *AGRP* and *POMC* anti-sense probes were linearized with PstI and transcribed from T7, and the sense probe was linearized with NotI and transcribed from SP6.

*In situ* hybridization was performed on the paraffin embedded sections with Digoxigenin-labelled probes diluted in hybridization mix. Each probe was hybridized overnight at 68–72°C in a Boekel slide incubator. After hybridization, slides were washed twice in 50% formamide, 50% 1xSSC and 0.1% Tween-20 at 65°C, then twice in MABT (0.1 M maleic acid, 150 mM NaCl, 0.1% Tween-20, pH 7.5) at room temperature. To block binding of nonspecific antibody, slides were incubated in 70% MABT, 20% natural sheep serum and 10% blocking reagent (Roche) for 2 hours. AP-conjugated anti-digoxigenin antibody (Roche) was diluted in this blocking solution at a dilution of 1/1500 and applied to slides, which were covered with parafilm and kept at room temperature overnight. Slides were subsequently rinsed in MABT 5 times for a minimum of 30 mins each. Slides hybridized with *POMC* probe were further rinsed in MABT overnight to reduce background. Slides were equilibrated in NTMT (100 mM NaCl, 50 mM MgCl_2_, 100 mM Tris pH 9.5, 0.1% Tween-20) twice for 10 mins each. To reveal colour, NBT-BCIP stock solution (Roche) was diluted in NTMT at a dilution of 1/1500 and applied to slides and covered with parafilm. The colour reaction of all probes, except for *POMC*, was performed at room temperature whereas the *POMC* colour reaction was conducted at 4°C.

The genes examined in our experiments have additional neuroendocrine functions as well as a putative function in pigmentation in the epidermis providing positive control of the anti-sense probe within sections [11,21,26,31,38–40]. Sections depicted in the figures are representative of staining between and within samples.

## Results

### Melanocyte distribution

To test whether melanocyte distribution may be responsible for embryonic quail pigmentation patterns we used *SOX10* as a melanocyte marker. *In situ* hybridization with a *SOX10* probe revealed that melanocytes are present in the epidermis and feather follicles at both E8 and E12 on the ventral and dorsal surface (Fig 1b). At both stages of development, there was *SOX10* staining both where melanin is present, as well as where it is absent. Thus melanocytes appeared to be evenly distributed throughout feather follicles regardless of their future pigmentation. Therefore, colour differences over the dorsum and ventrum of quail are unlikely to be due to differences in the distribution of melanocytes.

### Genes inhibiting MC1R in melanocytes

**Fig 2:**
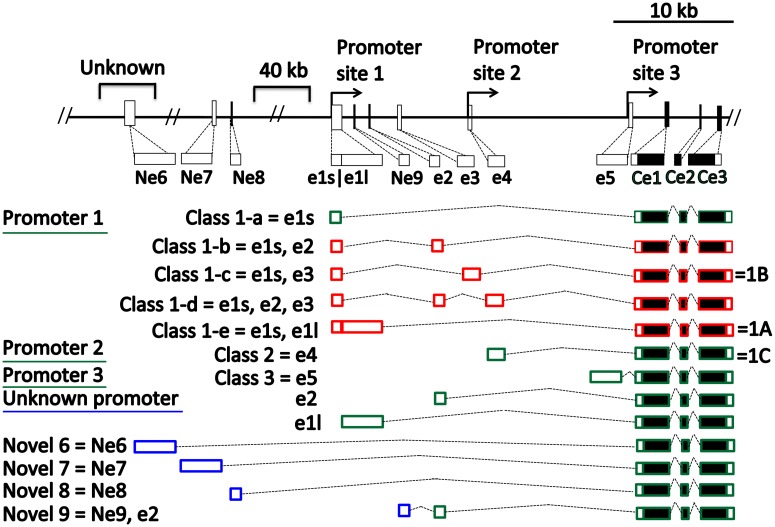
Correspondence of *ASIP* alternatively spliced transcripts between embryonic Japanese quail, adult quail and chicken. Empty boxes represent non-coding exons and solid boxes denote coding exons (Ce1-3). Green lines and green boxes represent promoter sites and transcripts, respectively, that were successfully amplified in quail embryonic development, whereas red denotes transcripts that were not amplified. Previously reported chicken transcript names begin with “Class” and the exons which they are comprised of begin with “e” and are listed in the middle [4]. Previously reported adult quail transcripts begin with “1” and are denoted to the right of the corresponding chicken transcript after the coding exons [4,21]. The names of the novel *ASIP* transcripts uncovered in this study begin with “Novel” and the exons they are comprised of are represented with blue boxes. The promoter site of the novel transcripts is unknown.

To test whether downregulation of the melanin synthesis pathway may be responsible for embryonic quail pigmentation patterns we investigated the patterns of expression of two genes encoding ligands that inhibit MC1R: *ASIP* and its paralogue *AGRP*. Previously, it has been reported that adult quail have three *ASIP* alternatively spliced transcripts (1a, 1b, 1c) [20], whereas the chicken has seven *ASIP* isoforms that are generated from three promoter sites (classes 1–3) [4,21]. Alignments of these *ASIP* isoforms reveal that there is close correspondence between them (Fig 2). There are some similarities between adult quail and chicken transcripts; chicken transcripts 1-c and 1-e are comprised of non-coding exon E1S alternatively spliced with non-coding exons E3 and E1L, respectively. In quail, transcripts with non-coding exons E3 and E1L correspond to adult quail 1b and 1A but are not alternatively spliced with E1S. In contrast, chicken class 2 and quail 1c, which include a single non-coding exon have good correspondence since they share high sequence similarity.

In RT-PCR experiments, we successfully amplified all previously described exons except for non-coding exon E3, and failed to amplify previously described alternatively spliced transcripts that combine more than one non-coding exon, (e.g. the leader exon E1S in class 1 transcripts) despite multiple attempts with numerous primer pairs. We report four new upstream non-coding exons and numerically name these following on from previously published exons. We present four novel *ASIP* alternatively spliced transcripts: Novel 6, Novel 7, Novel 8 and Novel 9 (Fig A in S1 File). Genbank searches against the *Gallus gallus* genome revealed that most of the novel exons (Novel 7–9) are located upstream of *ASIP* on chromosome 20, as expected, whereas quail novel 6 showed no hits against the chicken genome. Novel exons 7 and 8 are 40kb upstream of the *ASIP* promoter site 1 (Fig 2, Fig A in S1 File). Novel exon 9, is situated 30kb upstream of the first *ASIP* coding exon (e6), between E1L and E2 [4].

**Fig 3:**
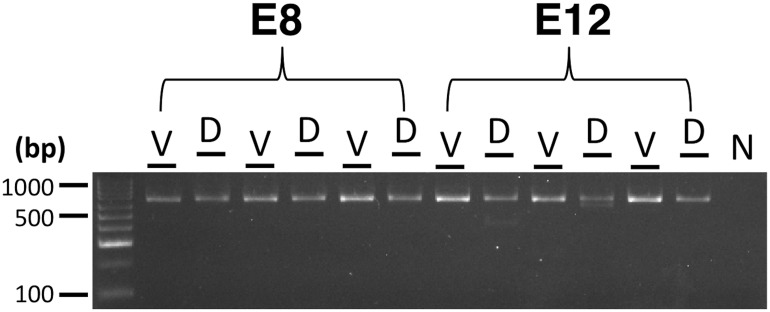
RT-PCR of the previously undocumented *ASIP* alternatively spliced transcript containing novel exon 7 in Japanese quail in three replicates per developmental stage. The expected amplicon length is 738 base pairs (Table A in S1 File). The developmental stage is above the gel lanes as well as body surface: V = Ventral, D = Dorsal. N = Negative control.

RT-PCR analyses revealed that the *ASIP* coding sequence was always expressed on both the dorsal and ventral surfaces and in both stages of quail embryonic development examined (Table 1). Most of the previously described exons as well as the novel exons were expressed on the ventral surface, albeit variably in some cases, at both E8 and E12 (Table 1). The exception was E1L, which was not expressed ventrally at E12. The expression of the exons on the dorsal surface was more variable than ventrally, and many were amplified in one developmental stage, but not the other (e.g. E2 at E8 but not E12) or not at all, such as E1L. There was also variation in the expression of the novel exons (Novel 8 and 10) within and between developmental stages. In contrast, novel exon 9 and 11 were expressed on both the ventral and dorsal surfaces in all embryos at stages E8 and E12 (Fig 3; Table 1).

**Table 1:** Patterns of exon expression for *ASIP* and *AGRP* in embryonic quail at developmental stage E8 and E12 determined by RT-PCR.

Target sequence		E8	E12
***ASIP* coding** **exons (6–8)**	Dorsal	+	+
	Ventral	+	+
**E1S**	Dorsal	-	+
	Ventral	+	+
**E1L**	Dorsal	-	-
	Ventral	+	-
**E2**	Dorsal	+	-
	Ventral	+	+
**E4**	Dorsal	+/-	-
	Ventral	+	+/-
**E5**	Dorsal	+	+/-
	Ventral	+	+
**Ne6**	Dorsal	+	+/-
	Ventral	+/-	+
**Ne7**	Dorsal	+	+
	Ventral	+	+
**Ne8**	Dorsal	+/-	-
	Ventral	+/-	+/-
**Ne9**	Dorsal	+	+
	Ventral	+	+
***AGRP* coding exons (1–2)**	Dorsal	+/-	-
	Ventral	+/-	+/-

“+” = present, “-” = absent, “+/-” = variable expression.

The second locus encoding a potential inhibitor of MC1R is *AGRP*. As there is no previous evidence implicating *AGRP* in pattern formation, or evidence for alternatively spliced transcripts, we focused on the expression patterns of the coding sequence. *AGRP* expression was detected at both developmental stages. Expression at E8 was variable on both the ventral and dorsal surfaces, whereas at E12, it was not expressed dorsally and had variable expression ventrally (Table 1).

**Fig 4:**
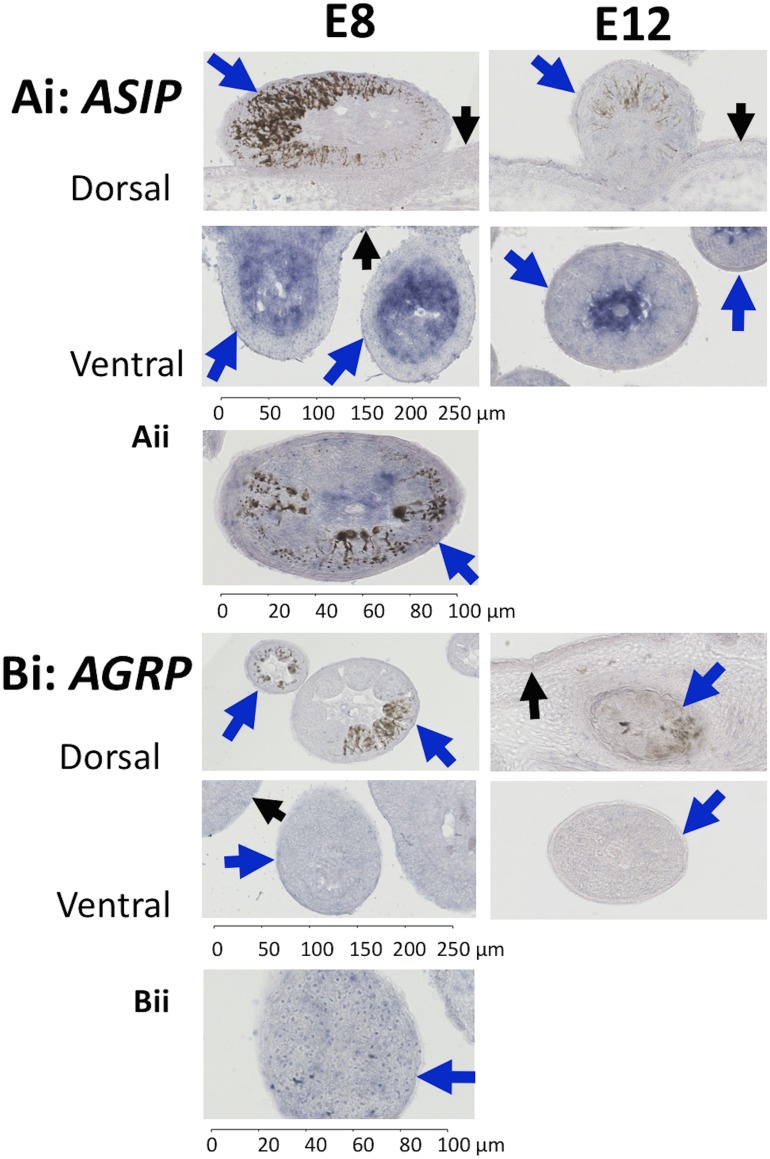
*In situ* hybridization results for *ASIP* and *AGRP* in quail feather follicles at E8 and E12. The epidermis is labelled with a small black arrow and feather follicles with a large blue arrow. Ai) *ASIP* is expressed in the dermal pulp of ventral feather follicles in E8 and E12 embryos, but is not visible on the dorsal surface. Bi) *AGRP* was not expressed in the feather follicles in early or late development, either ventrally or dorsally. Aii) *ASIP* expression in a feather follicle with eumelanin on the flank of a Japanese quail embryo at E12. In some feather follicles in the flank or on the wing of E12 embryos *ASIP* expression was observed with eumelanin pigmentation. Bii). Representative transcription of *AGRP* in ventral developing feather follicles in E8 Japanese quail embryos. Similar staining was found in feather follicles on the dorsal surface of quail E8, as well as both the ventral and dorsal surface of quail E12, and the epidermis.

To examine whether *ASIP* and *AGRP* are expressed within feather follicles during development we used *in situ* hybridization. *ASIP* is expressed early in development at E8 and this expression continued into later stages of development at E12 (Fig 4Ai). In both early and late stages of development, *ASIP* is strongly expressed throughout the pulp of developing feather follicles on the ventral surface and many ventral cells surrounding the dermal pulp in the epidermis exhibit weak expression of *ASIP*. Some feather follicles on the flanks of the ventral surface near or on the wing have eumelanin pigmentation and a minority of these feather follicles had some staining for *ASIP* in the feather pulp both where eumelanin is present as well as where it is absent (Fig 4Aii). In contrast, in feather follicles on the dorsal surface of E8 and E12 embryos, there was no staining for *ASIP* in the dermal pulp but there does appear to be faint expression within some cells of the feather pulp and the epidermis. The pattern of expression of *ASIP* on the dorsal surface did not vary between feather follicles that are pigmented or unpigmented.

Staining of *AGRP* was less prevalent than *ASIP* (Fig 4). Unlike *ASIP*, *AGRP* was not strongly expressed in the pulp of developing feather follicles on the dorsal or ventral surface, at either stage of development or in the epidermis. However, weak expression of *AGRP* was commonly observed on the ventral and dorsal surface of E8 as well as E12 in feather follicles both with and without melanin (Fig 4bii).

### Genes activating MC1R in melanocytes

**Fig 5:**
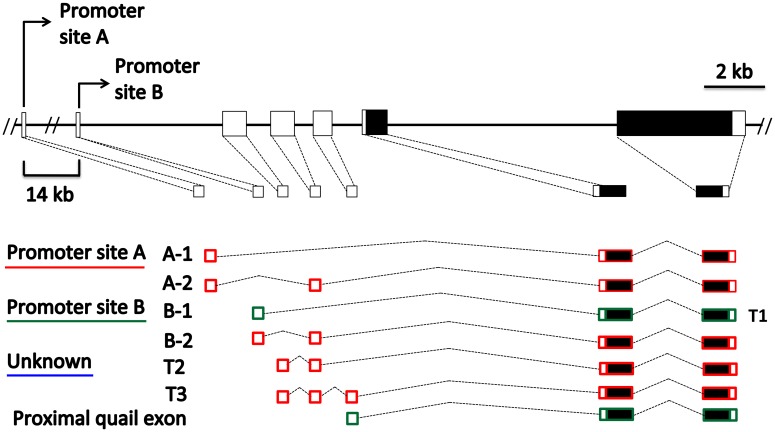
Correspondence between alternatively spliced transcripts of *POMC* in embryonic and adult Japanese quail, and chicken. Chicken promoter sites and alternatively spliced transcripts are listed on the left, and the corresponding quail transcript is listed to the right of the transcript. Quail *POMC* transcripts that have no correspondence with chicken transcripts are listed with the novel promoter site [5]. Empty boxes represent non-coding exons and solid boxes denote coding exons. The previously described non-coding exons that were amplified in quail embryonic development are represented with green, whereas red denotes the combination of promoter site and non-coding exons that we were unable to amplify. The promoter sites for T2, T3 and the proximal quail exon are unknown.

We investigated three loci that are involved in generating melanocortin ligands that activate MC1R: *POMC*, *PCSK1* and *PCSK2*. Yoshihara et al. (2011) previously described four transcripts of *POMC* in chicken transcribed from two promoter sites with or without an additional non-coding exon: A-1, A-2, B-1, and B-2. The chicken distal promoter site A is located 14kb upstream in another gene, *Angptl7*, whereas promoter site B is located within the *POMC* gene. In addition, three unpublished adult quail *POMC* alternatively spliced transcripts are available on NCBI: T1, T2, and T3. Quail T1 has promoter site B while quail T2 and T3 have a leader exon not found in chicken, and none contain chicken promoter site A (Fig 5; Table A in S1 File) [5]. Thus, there are distinct differences between quail and chicken transcripts.

RT-PCR analyses revealed that the coding exons of *POMC* are always expressed on both the dorsal and ventral surfaces in quail E8 and E12 (Table 2). The chicken *POMC* non-coding exon *POMC* A-1 and quail T2 could not be amplified despite multiple attempts with numerous primer pairs (Fig 5; Table A in S1 File). Transcripts arising from promoter site B were present in almost all ventral and dorsal samples, at both E8 and E12, the exception being one dorsal sample in E8. The proximal quail non-coding *POMC* exon was also variable in its expression, as it was amplified in two samples from each of E8 and E12, and was expressed in both ventral and dorsal samples.

**Table 2:** Expression of *POMC*, *POMC* alternatively spliced exons, *PC1* and *PC2* in quail at embryonic stage E8 and E12 determined by RT-PCR.

Target sequence		E8	E12
***POMC* coding exons**	Dorsal	+	+
	Ventral	+	+
***POMC* promoter B**	Dorsal	+/-	+
	Ventral	+	+
***POMC* proximal quail non-coding exon**	Dorsal	+/-	+/-
	Ventral	+/-	+/-
***PCSK1 coding exons***	Dorsal	+	+
	Ventral	+/-	+
***PCSK2 coding exons***	Dorsal	+	+
	Ventral	+	+

“+” = present, and “+/-” = variable expression.

**Fig 6:**
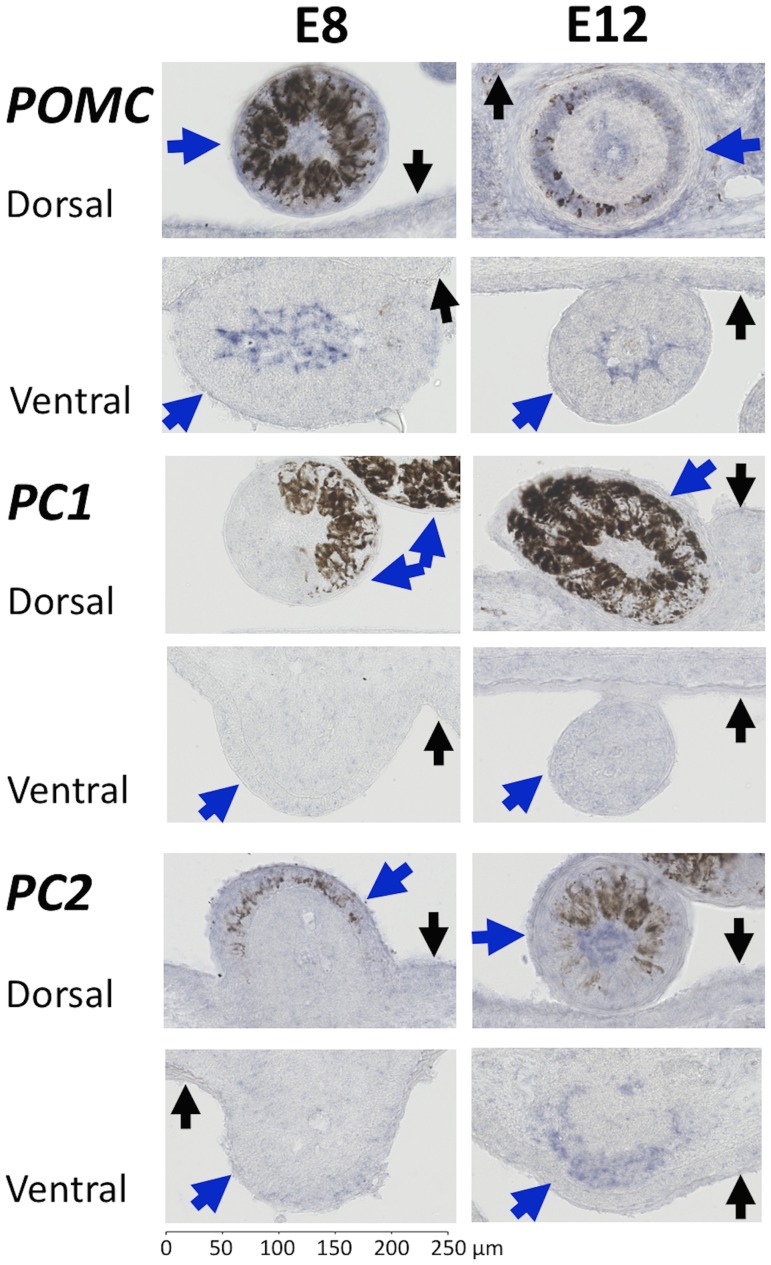
Patterns of expression of *POMC*, *PC1* and *PC2* in feather follicles at E8 and E12. The epidermis is labelled with a small black arrow and feather follicles with a large blue arrow. *POMC* is expressed in the dermal pulp of ventral and dorsal feather follicles in E8 and E12 embryos. Weak expression of *PC1* was observed in developing feather follicles but did not develop further at E12. There is weak transcription of *PC2* at E8 on both ventral and dorsal surfaces, but when feather development is more progressed (e.g. E12), *PC2* is strongly expressed in feather follicles on both the ventral and dorsal surface.

*In situ* hybridization revealed that *POMC* is strongly expressed in the epidermis and feather follicles on the dorsal and ventral surface of E8 and E12 embryos (Fig 6). Within feather follicles, *POMC* is apparent in the feather cuticle as well as the feather pulp on both the ventral and dorsal surface, where both eumelanin and pheomelanin are present. Thus, *POMC* may be required for melanin synthesis regardless of melanin type.

*PCSK1* and *PCSK2* encode endoproteases that cleave *POMC* products to make ACTH and MSH, respectively. As there is no evidence to suggest that there are alternatively spliced transcripts of *PCSK1* and *PCSK2* that have a role in feather follicle pigmentation, we focused on the expression patterns of the coding sequence. RT-PCR revealed that *PCSK1* was present in nearly all samples on the ventral and dorsal surface at E8 and E12, the exception being one ventral sample from E8 (Table 2). Similarly, *PCSK2* was present in all dorsal and ventral samples, in both stages of development examined.

Examining where these genes are expressed, *in situ* hybridization demonstrated weak expression of *PCSK1* within the feather pulp and the feather cuticle on the ventral and dorsal surface in E8 quail embryos (Fig 6). At E12 the low level of expression of *PCSK1* did not progress further. *PCSK2* is faintly expressed in feather follicles and the epidermis at E8 on both ventral and dorsal surfaces. At E12, *PCSK2* was also faintly expressed in the epidermis but there was strong expression of *PCSK2* within the feather pulp as well as the feather cuticle (Fig 6). The patterns of expression were consistent in feather follicles over both the ventral and dorsal surface where eumelanin and pheomelanin are present.

## Discussion

Our data provide some of the first evidence that extracellular ligands involved in activating MC1R may be involved in plumage patterning in quail embryogenesis, which has implications for pigmentation patterns in birds. *SOX10* staining demonstrated that the distribution of melanocytes cannot account for pattern formation. Therefore, the generation of pigmentation patterns is likely due to regulation of melanin synthesis. We discovered novel quail *ASIP* transcripts suggesting variation in the regulation of MC1R inhibition in comparison to adults [4,21]. As in previous studies, we found support for the association of the pale-bellied phenotype of quail with *ASIP* expression, indicating likely conservation of the mechanism of a pale ventrum between mammals and birds [3,5,15–20]. Conversely, unlike the situation in mice, we found little support for a role of *ASIP* in the regulation of dorsal temporal-specific patterning in feathers. Of the six *POMC* transcripts previously described, only one has been conserved between quail and chicken. *POMC* transcripts showed variable expression over the dorsum and ventrum. However, *POMC* is expressed within both dorsal and ventral feather follicles indicating that activation of MC1R by melanocortins may be required for eumelanin production. Of the POMC endoproteases, only *PCSK2* is expressed within feather follicles late in quail embryogenesis during feather growth. Therefore, it may be that *POMC* products are directly cleaved by *PCSK2* to make α-MSH and that α-MSH is the main melanocortin activating the MC1R pathway at this developmental stage in birds. Together, this suggests that MC1R is differentially stimulated across the ventral and dorsal surfaces to create variation in plumage coloration.

*Sox10* is a marker for all cells of neural crest origin including melanocytes. This should not be an issue in this study since we focused here on epidermis and feather follicles, where non-melanocyte neural crest derivatives are absent.

There was little support for a role of *AGRP* in ventral pigmentation or within-feather patterning (Fig 4Bi). RT-PCR results demonstrated that *AGRP* is variable in expression over the ventral and dorsal surface of developing quail embryos, which would be consistent with a temporal specific function (Table 1). Although we found weak expression of *AGRP* in E8 feather follicles, expression was weaker or absent by E12 (Fig 4Bii). If *AGRP* has a ventral specific function in inhibiting MC1R the expectation would be that it would have been expressed in all ventral samples, but this was not the case (Table 1).

There were differences in the expression of the seven previously described alternatively spliced transcripts of *ASIP*, between adult quail, chicken and embryonic quail [4,21]. Of the seven transcripts, only three were observed in embryonic quail, and only one of these three (E4) has previously been reported in adult quail. In mice and rabbits, the distal *ASIP* promoter leads to ventral specific coloration, and the proximal promoter performs temporal control of coloration during hair development [16–18,20]. The chicken distal promoter (Class a) is expressed both dorsally and ventrally in adult quail indicating that the putative distal *ASIP* promoter is not ventral specific in quail. Of the transcripts expressed in quail embryonic development the *ASIP* non-coding exon E1L is ventral specific, as in the chicken but unlike adult quail. One exon present in adult quail and chicken, E3, could not be amplified in embryogenesis (Fig A in S1 File).

We report four undescribed 5’ non-coding exons of *ASIP*, three of which have homologous sequences on the chicken genome (Fig 2; Fig A in S1 File), whereas the precise location of Novel 6 is unknown. The exon found in Novel 9 is positioned just upstream of exon E2, to which it is spliced, whereas the novel exons 7 and 8 are located ~65kb upstream of the *ASIP* coding exons. As our intention was to document the distribution of existing *ASIP* transcripts between embryonic quail, adult quail and the chicken, we did not perform 5’ RACE that would clarify *ASIP* transcripts, and which promoter sites initiate transcription. Nevertheless, our findings indicate surprising variation in the expression of *ASIP* alternatively spliced transcripts between embryonic quail, adult quail, and chicken breeds, that suggest both variation among developmental stages and between species. The finding of additional non-coding exons ~40kb upstream of known exons suggest regulation of *ASIP* expression at least as complex as that in mice.

*In situ* hybridization with *AGRP* and *ASIP* failed to demonstrate strong expression of these genes on the dorsal surface of embryos, in contrast to RT-PCR results. Variation between RT-PCR and *in situ* hybridization results may be due to a difference in the sensitivity of these techniques (Fig 4Bii; Table 1). However, a difference in the sensitivity of techniques is unlikely to be the cause of a discrepancy in the case of *AGRP* given that the expression of the *AGRP* coding sequence was variable within and between samples (Table 1). Unlike *AGRP*, RT-PCR results for the coding region of *ASIP* demonstrated consistent expression on both the dorsal and ventral surface of E8 and E12 embryos, yet *in situ* hybridization indicated that *ASIP* was strongly expressed within developing feather follicles only on the ventral surface. The most likely explanation for this discrepancy is the presence of *ASIP* expression in the epidermis, which was included in the RT-PCR experiments, but we cannot rule out the possibility that weak *ASIP* expression does occur in dorsal feather follicles.

*POMC* appears to have few alternatively spliced transcripts in quail embryos (Figs 2 and 5; Table 2; Table A in S1 File). Similar to *ASIP*, we found that *POMC* transcripts varied in their patterns of expression between quail embryonic development and the chicken. Of the *POMC* transcripts expressed, none were dorsal specific. However, this may be due to a lack of detailed 5’ information. A future study could investigate *POMC* alternatively spliced transcripts and promoter sites utilizing RNASeq or 5’ RACE.

Our main finding, that melanocytes are present in all feather follicles and that loci involved in the production of melanocortin ligands that activate MC1R (*POMC* and *PCSK2*) are transcribed in feather follicles on the dorsal surface, indicates that melanin synthesis and the generation of pigmentation patterns may require MC1R activation. Chicken MC1R expressed *in vitro* has low basal activity (measured by cAMP production) in the absence of ligands [41]. Chicken MC1R has a much lower binding affinity for MSH than human MC1R, e.g. the binding affinity of α-MSH to chicken MC1R is 363 nM compared to 0.210 nM in humans [11]. A low density of MC1R receptor number may result in lower endogenous signaling [15]. The density of MC1R receptors in bird plumage is currently unknown, and experimental downregulation of POMC in birds has not been performed. Nevertheless, given the low basal activity of MC1R in chicken in comparison with mice it may be that *POMC* is required for MC1R activation in birds [11,15,41].

Among the prohormone convertase genes, *PCSK1* was weakly expressed in comparison to the positive control, with some inconsistency between RT-PCR and *in situ* hybridization results probably due to expression of this gene in the epidermis (Fig 6; Table 2). *PCSK2* showed clear patterns of expression on both dorsal and ventral surfaces, particularly at E12. Since PC2 is required for generation of α-MSH, from either POMC or ACTH, this strongly suggests that α-MSH is a more important ligand than ACTH in quail, contrary to a suggestion that ACTH might be more important because of its higher binding affinity to MC1R in chicken [41]. Our results are in contrast with a study that found expression of *PCSK1*, *PCSK2* and *POMC* in chicken feather follicles [5] but similar to a study that found no detectable expression of *PCSK1* in the growing feathers of barn owls (*Tyto alba*) [42]. However, the interpretation is complicated since the two breeds are apigmented (Silky chicken) and barred (Okayama-Jidori), and there was no comparison made between the patterns of gene expression between these breeds.

Previous studies emphasized a role of inhibition of MC1R for within-feather pattern formation [2,4,21]. For example, in adult chicken, *ASIP* expression is found only adjacent to pheomelanin or apigmented areas [2,4]. In contrast with these studies, our system comprised controlled stages of embryogenesis in a wild-type phenotype and we found that *ASIP* was distributed throughout the feather pulp, but only on the ventrum (Fig 4Ai). Similar to previous findings, *POMC* and *PCSK2* were found to be expressed throughout feather follicles, but in contrast we did not detect *PCSK1* within feather follicles [4] (Fig 6). It is interesting that *ASIP*, *POMC* and *PCSK2* are expressed within feather follicles of quail embryos on the ventral surface but quantification of ASIP and MSH peptides in quail embryos is unknown. We showed that activation of MC1R may be required for eumelanogenesis on the ventral and dorsal surface, but it is unknown what mechanism may inhibit MC1R to create the species typical dorsal stripes and within-feather pigmentation. Given that there is a temporal delay of melanocyte development on the ventral surface compared to the dorsal surface, the genes of interest may potentially act on the species typical dorsal stripes prior to E8 (Fig 1).

Several other genes may be involved in regulating MC1R activity to create dorsal stripes and within-feather patterning in quail. For example, β-defensin, which is a peptide that is structurally similar to ASIP, is highly polymorphic in sequence and copy number and has the potential for extensive cross-talk with the melanocortin system in vertebrates [43]. Alternatively, in mice *Corin* appears to suppress the *ASIP* pathway and modulates temporal banding patterns [44], but does not appear to have a similar function in cats [19]. Attractin prevents follicular melanocytes from responding to ASIP, and loss of attractin leads to production of little or no yellow pigmentation [45]. Similarly, mice that lack the intracellular ubiquitin ligase mahogunin also fail to respond to ASIP [46]. Thus, there are many other loci that could differentially regulate the MC1R pathway to produce dorsal between-stripe and within-feather patterning.

It is thought that plumage patterns may be a result of a reaction-diffusion mechanism [47], but given that this relies on an activator that upregulates its own inhibitor, it is unclear how molecular mechanisms of pigmentation in birds might relate to this model. We found little evidence for a pigmentation function of *AGRP* whereas our data are consistent with previous reports that, that across vertebrates *ASIP* plays an important role in controlling receptor activity. Quail may require *MC1R* activation for eumelanogenesis in dorsal feathers indicating that the response of MC1R to activating ligands via *POMC* and *PCSK2* may depend on the genetic background of vertebrate systems and may be species or system specific.

## Supporting information

S1 File **Fig A. New *Asip* alternatively spliced transcripts that have not been previously documented in the avian literature**. The transcript name is given followed by the name of the new non-coding exon. Novel 6 (Accession—KR873138) had no significant similarity with the chicken genome. The accession number for the other *Asip* novel transcripts are as follows: Novel 7—KR873139, Novel 8—KR873140 and Novel 9—KR873141. Quail transcripts (Cj: *Coturnix japonica*) are aligned to the chicken genome (Gg; *Gallus gallus*). A vertical bar—|—indicates matching base pairs and a space indicates no match, whereas dashes indicate indels. Novel 9 contains a new leader exon alternatively spliced with the existing chicken non-coding exon E2 (Yoshihara et al. 2012) that is highlighted in grey. **Table A. Primers used for amplifying mRNA transcripts**.
(PDF)
