# Supplementary material for: The differential expression of MC1R regulators in dorsal and ventral quail plumages during embryogenesis: Implications for plumage pattern formation
Source: PLoS One. 2017 Mar 29;12(3):e0174714. doi: 10.1371/journal.pone.0174714 (PMC5371383; doi:10.1371/journal.pone.0174714)
Supplement: S1 File — Fig A. New Asip alternatively spliced transcripts that have not been previously documented in the avian literature. The transcript name is given followed by the name of the new non-coding exon. Novel 6 (Accession—KR873138) had no significant similarity with the chicken genome. The accession number for the other Asip novel transcripts are as follows: Novel 7—KR873139, Novel 8—KR873140 and Novel 9—KR873141. Quail transcripts (Cj: Coturnix japonica) are aligned to the chicken genome (Gg; Gallus gallus). A vertical bar—|—indicates matching base pairs and a space indicates no match, whereas dashes indicate indels. Novel 9 contains a new leader exon alternatively spliced with the existing chicken non-coding exon E2 (Yoshihara et al. 2012) that is highlighted in grey. Table A. Primers used for amplifying mRNA transcripts. (PDF) [file pone.0174714.s001.pdf]

The differential expression of MC1R regulators in dorsal and ventral quail plumages during embryogenesis: implications for plumage pattern formation

Thanh-Lan Gluckman and Nicholas I Mundy.

#Novel 6

```
CAACGAGCTACCTTCAGGTGGTGTGAGGCTGGCAATGGGGTGCAGATGGGGCCTCATATCTC
TGAGTAAATGGAACAGGCAGATGAGAAGGGCACAGCTGTGCTCTTCCACCTCAGCATGCTCC
CTGGACATCCATGCCTCTTGGCTGCCCACTAACCTCAGCTGGAGAGTGCACAGTGATTAATA
TTAGTGAATCTGATCTAAGCCTATTGCAAACCATTGATCTGAGCGGGCTGCACTGCTCCAAA
AATAGCCTCACAATATTACTGAGGCATAGCTCTTGGTGTGGTGAGCTGCTCAAGCTGCCTAA
TCACATACAAGACCCCAGGCTCTGTATGTTCTCAATGCTTTTTTCCTGTGACCACTGCTCAT
CACACTTGGCTACTGTGGGTTAATACAGCTATGTCATGTTCAGCCAAATCAGCTGTCACATC
AGCTCTCCATTT
```

#Novel 7

```
Cj CAGTGACTACATCTGGAAAAGAAAAAAGAAGAAACATGAAGATGACCATACGCCGTTGTA
   ||||||| |||||||||||||||||||  |||||||||||||||||| ||||| ||||||
Gg CAGTGACAACATCTGGAAAAGAAAAAA--AGAAACATGAAGATGACCGTACGCTGTTGTA

Cj TTTCCTTCTCAGATGAGTTATATTTTGAAAGCAGCAATAAAAAA-TTCTCAGATAAGGGA
   |||||||||||||||||||||||||||||||||||||||||||| ||||| | |||||||
Gg TTTCCTTCTCAGATGAGTTATATTTTGAAAGCAGCAATAAAAAACTTCTCGGGTAAGGGA

Cj TTGGAGAAGTGATGGATTTGTGGCAGCGGCTGTCTTTTGGTCCTTCGTTGACTGCTTTGG
   |||||||||||||||| |||||||||||||||||||||||||||| | |||||  | |||
Gg TTGGAGAAGTGATGGAATTGTGGCAGCGGCTGTCTTTTGGTCCTTGGCTGACTATTCTGG

Cj GGTTGA--ATTTTTCTTTTTAATGTTCACATGTGTGTGCCTTTTTTGGGGGAAATGGGGC
   ||||||   ||||||||||| ||||||||| |||||| |||||||| ||| |||||||||
Gg GGTTGATTTTTTTTCTTTTTGATGTTCACACGTGTGTACCTTTTTT-GGGAAAATGGGGC

Cj CAGATTCCCCCCTGGCTGAGAGTCTACAGCTTCTTAAAGGTGAAGGCTGTTCCAGT
   |||||||||||||||||||||| |||||||||||||||||||||||||||||||||
Gg CAGATTCCCCCCTGGCTGAGAGGCTACAGCTTCTTAAAGGTGAAGGCTGTTCCAGT
```

#Novel 8

```
Cj TTGTGGCAGCGGCTGTCTTTTGGTCCTTCATTGACTGCTTTGGGGTTGA--ATTTTTCTT
   ||||||||||||||||||||||||||||   |||||  | |||||||||   ||||||||
Gg TTGTGGCAGCGGCTGTCTTTTGGTCCTTGGCTGACTATTCTGGGGTTGATTTTTTTTCTT

Cj TTTAATGTTCCACCATGTGTGTGCCTTTTTTGGGGGAAATGGGGCCAGATTCCCCCCTGG
   ||| ||||||   || |||||| |||||||| ||| ||||||||||||||||||||||||
Gg TTTGATGTTC--ACACGTGTGTACCTTTTTT-GGGAAAATGGGGCCAGATTCCCCCCTGG

Cj CCTGAGAGTCTACAGCTTCTTAAAGGTGAAGGCTG-TCCAGT
    ||||||| |||||||||||||||||||||||||| ||||||
Gg -CTGAGAGGCTACAGCTTCTTAAAGGTGAAGGCTGTTCCAGT
```

#Novel 9, includes e2

```
Cj TTCCAGGACCAGCAACTCTTATTTATAAATGCTATACAGCGTATATGTTAAGATTTATAC
   |||||| ||||||| |||||||||||||||| ||||| ||||||||||| ||||||||||
Gg TTCCAGAACCAGCAGCTCTTATTTATAAATGTTATACCGCGTATATGTTCAGATTTATAC

Cj AAGATGTTTTCATGGTACGCATAGAATTCTGAACCCTGTAAACACACTGATGGCATTAAC
   |||||||||||||||||||||||||||||||||||||||||||||| |||||||||||||
Gg AAGATGTTTTCATGGTACGCATAGAATTCTGAACCCTGTAAACACATTGATGGCATTAAC

Cj AAGGACCAGATGTCAGTTGCCTTTCTCATAAAGAAAACAACAAG
   |||||||||||||||||||||||||||||| |||||||||||||
Gg AAGGACCAGATGTCAGTTGCCTTTCTCATATAGAAAACAACAAG
```

Fig A. New *Asip* alternatively spliced transcripts that have not been previously documented in the avian literature. The transcript name is given followed by the name of the new non-coding exon. Novel 6 (Accession - KR873138) had no significant similarity with the chicken genome. The accession number for the other *Asip* novel transcripts are as follows: Novel 7 - KR873139, Novel 8 - KR873140 and Novel 9 - KR873141. Quail transcripts (Cj: *Coturnix japonica*) are aligned to the chicken genome (Gg; *Gallus gallus*). A vertical bar - I - indicates matching base pairs and a space indicates no match, whereas dashes indicate indels. Novel 9 contains a new leader exon alternatively spliced with the existing chicken non-coding exon E2 (Yoshihara et al. 2012) that is highlighted in grey.

Table A. Primers used for amplifying mRNA transcripts. The number of exons out of the total number of coding exons is provided as well as the expected sequence length, source of primers, and the accession number of each transcript.

Target sequence	Primer	Primer sequence	Non-coding exons (bp)	Coding exons (bp)	Expected amplicon length (bp)	Primer source	Accession
β-actin							
Coding sequence	ACT1F	TGCGTGACATCAAGGAGAAG	-	Exon 2/2: 250	250	Nadeau 2006	AB199913.1
	ACT1R	CAGGTCCTTACGGATGTCCA				Nadeau 2006	
Asip							
Coding sequence	AsipF2	TCATTTTCATGACAGTGGGATT	-	Exon 1-3/3: 465	450	Nadeau 2006	NM_001115079.1
	AsipR2.0	CCTTAACATGTTCCTCATTAGGTTTA				Designed	
E1S	E1SF	TGAAAAGGAAGCAGAACCAGA	58	Exon 1-3/3: 465	523	Designed	AB518061.1
	AsipR2.0	CCTTAACATGTTCCTCATTAGGTTTA				Designed	
E1L	E1LF	AGTTTTGGAGGTTCATTTCTAATGT	405	Exon 1-3/3: 465	870	Designed	AB518065.1
	AsipR2.0	CCTTAACATGTTCCTCATTAGGTTTA				Designed	
E2	E2F	TAAACACATTGATGGCATTAACAA	64	Exon 1-3/3: 465	529	Designed	AB518062.1
	AsipR2.0	CCTTAACATGTTCCTCATTAGGTTTA				Designed	
E3	E3F	GAAGCAGGCAGTCTTCTTGG	72	Exon 1-3/3: 465	470	Designed	AB518063.1
	AsipR2.0	CCTTAACATGTTCCTCATTAGGTTTA				Designed	
E4	AsipF8	CCAGCATTTTCATATTTTCTGGA	111	Exon 1-3/3: 465	576	Nadeau 2006	AB518066.1
	AsipR2.0	CCTTAACATGTTCCTCATTAGGTTTA				Designed	
E5	E5F	TGAAATCAGTTGTGGCAGGAA	189	Exon 1-3/3: 465	654	Designed	AB518067.1
	AsipR2.0	CCTTAACATGTTCCTCATTAGGTTTA				Designed	
Novel 6	NewE1F	GTGTGGTTGTGATGGTGATGG	446	Exon 1/3: 131	577	Designed	KR873138
	NewE1R	GGGAGATCTGGGAGGTTCATT				Designed	
Novel 7	NewE4F	GAGATCTTAAACAGCGCTGCA	293	Exon 3/3: 445	738	Designed	KR873139
	E2_R	CAGCCTTAACATGTTCCTCATTA				Designed	
Novel 8	NewE1F	GTGTGGTTGTGATGGTGATGG	158	Exon 1/3: 131	289	Designed	KR873140

	NewE1R	GGGAGATCTGGGAGGTTCATT				Designed	
Novel 9	NewE3F	TTTTTGGGAGCTGTTGTCCTC	164	Exon 3/3: 445	669	Designed	KR873141
	E2_R	CAGCCTTAACATGTTCCTCATTA				Designed	
Agrp							
Coding sequence	AgrpF1	CCAGGACCATGCTGAAC	-	Exon 2/2: 449	449	Nadeau 2006	AB489990.1
	AgrpR	CAGGAAGATCAGCACCACCT				Designed	
Pomc							
Coding sequence	PomcF	CTGGGGCTGCTGCTGCTGTGT	-	Exon 2/2: 717	717	Designed	NM_001031098.1
	PomcR	TGACCCTTCTTGTAGGCGCTTT				Designed	
Promoter A	APomc	CCCATAAGCGACTTGCCTTC	169	Exon 2/2: 711	880	Designed	AB593424
	QER	CAGAGTCATCAGCGGGGTCT				Designed	
A-2; B-2	Pomc B-2	CTCTCCCCCTGCAGCATC	143	Exon 2/2: 711	854	Designed	AB593425; AB593427
	QER	CAGAGTCATCAGCGGGGTCT				Designed	
Promoter B	BF	AGCGCTCCTCTGCAGTTTG	42	Exon 2/2: 711	753	Designed	AB593426.1
	BR	CAGAGTCATCAGCGGGGTCT				Designed	
T2	QEF2	ACTTCCAGCGTCTCCCAGAG	252	Exon 2/2: 711	963	Designed	AB620012.1
	QER	CAGAGTCATCAGCGGGGTCT				Designed	
T3	QEF1	GATTTCGGAGGCAAAGGATG	175	Exon 2/2: 711	886	Designed	AB620013.1
	QER	CAGAGTCATCAGCGGGGTCT				Designed	
PCSK1							
Coding sequence	Pc1F	CTACGCCAACTATGATCCAAGG	-	Exon 5-10/14: 847	847	Designed	XM_003643060.2
	Pc1R	TTTCCATCTTTTGGGATCAGC				Designed	
PCSK2							
Coding sequence	Pc2F	GGGAGGGAAAGGAAGCATCT	-	Exon 9-11/12: 800	800	Designed	XM_419332
	Pc2R	GGTCTTCTCCCCAAGTGTGTG				Designed	

Reference

Yoshihara, C., Fukao, A., Ando, K., Tashiro, Y., et al. (2012) Elaborate color patterns of individual chicken feathers may be formed by the agouti signaling protein. *General and Comparative Endocrinology*. 175 (3), 495–499.
